# Multi-method validation of the new computerized test of fluid intelligence MatriKS

**DOI:** 10.3758/s13428-026-03049-2

**Published:** 2026-06-08

**Authors:** Debora de Chiusole, Ottavia M. Epifania, Pasquale Anselmi, Andrea Brancaccio, Noemi Mazzoni, Matilde Spinoso, Matteo Orsoni, Sara Giovagnoli, Irene Pierluigi, Alice Bacherini, Mariagrazia Benassi, Giulia Balboni, Luca Stefanutti

**Affiliations:** 1 Department of Philosophy, Sociology, Education and Applied Psychology, University of Padua, Via Venezia 14, 35131 Padova, Italy; 2 Department of Psychology and Cognitive Science, University of Trento, Corso Bettini 84, 38068 Rovereto, Italy; 3 Department of Psychology “Renzo Canestrari”, University of Bologna, Piazza Aldo Moro 90, 47521 Cesena, Italy; 4 Department of Theoretical and Applied Sciences, eCampus University, Via Isimbardi 10, 22060 Novedrate (CO), Italy; 5 Department of Philosophy, Social Sciences and Education, University of Perugia, Piazza G. Ermini 1, 06123 Perugia, Italy

**Keywords:** Knowledge space theory, Fluid intelligence, Multi-method validation, Matriks, Raven’s matrices

## Abstract

This paper introduces MatriKS, a new computerized tool for the assessment of fluid intelligence based on Raven-like matrices. Based on knowledge structure theory (KST), a mathematical framework initially designed for efficient assessment and personalized learning, MatriKS is the first large-scale application of KST to fluid intelligence assessment. The validation results for MatriKS, suitable for Italian individuals aged 4 to 11 ($N = 568$), are presented. A multi-method approach incorporating classical test theory (CTT), item response theory (IRT), and KST was employed. Each of the three approaches, with its own assumptions and models, highlights structural properties of the data that are not captured by the other two. Nevertheless, the three approaches provide an acceptable modeling of the data supporting the adequate functioning of MatriKS. The study concludes by exploring the methodological and practical benefits of using KST for constructing tests and estimating individual cognitive profiles.

**Supplementary Information:**

The online version contains supplementary material available at 10.3758/s13428-026-03049-2.

## Introduction

It is not new that the advancement of science is strictly dependent on newer, more sophisticated techniques to measure and analyze the variables under investigation. This is particularly true for psychology, a century-and-a-half -old discipline that is inherently complex, with multiple components, most of which are latent. As in other empirical sciences, *multi-method measurement* allows researchers to explore different dimensions and perspectives of a scientific phenomenon. This approach can provide a more comprehensive understanding of psychological constructs and offer stronger evidence for psychological theories than single-method studies. Moreover, researchers can cross-verify their findings by comparing results across different methods, reducing the likelihood of errors or biases associated with a single approach. This enhances the credibility and reliability of the conclusions drawn from the research and contributes to the validity and robustness of a research study (see, e.g., Eid & Diener, 2006).

In psychology, at least three different measurement approaches are available nowadays: classical test theory (CTT; see, e.g., Lord & Novick, 1968; Novick, 1966), item response theory (IRT; see, e.g., Embretson & Reise, 2000) and knowledge structure theory (KST; see, e.g., Doignon & Falmagne, 1999; Falmagne, Albert, Doble, Eppstein, & Hu, 2013; Heller & Stefanutti, 2024). Among the three approaches, KST is the newest and least known.

In the field of knowledge assessment and learning, KST marks a significant departure from traditional psychometric theories. Indeed, KST is not intended to quantify knowledge or learning in a conventional numerical sense. Rather, it focuses on identifying the specific items (or skills) a person masters within a given field of knowledge at a given moment. Thus, the feedback of a KST-based assessment is nonnumerical, assuming the form of a set.

Contrarily to KST, CTT and IRT have a long tradition in the field of test and questionnaire validation, and are commonly used for this purpose. While CTT focuses on classical measures such as test scores, IRT offers a more nuanced analysis by considering item-level properties and latent traits. It is not uncommon to see these two methods used together for comprehensive test validation (see, e.g., Bacherini, Anselmi, Havercamp, & Balboni, 2024; Bechger, Maris, Verstralen, & Béguin, 2003; Eluwa, Eluwa, & Abang, 2011). CTT was originally conceived as an operational theory of the true score in a psychological test or questionnaire (Lord & Novick, 1968). Later on, it was extended to approaches for latent variables, such as factor analysis or structural equation modeling. On the other hand, both IRT and KST originated as latent variable theories, which clearly separate an observed score from a true latent variable. Specifically, IRT models the latent variable as a continuous trait, whereas in KST it is a discrete state.

Until a few years ago, KST has been applied almost exclusively to the educational context (see, e.g., Falmagne et al., 2013). In the last decade, some extensions of KST have been proposed that moved it from its native landscape to the wider field of psychological (Spoto, Stefanutti, & Vidotto, 2010; Bottesi, Spoto, Freeston, Sanavio, & Vidotto, 2015; Donadello et al., 2017) and neuropsychological assessment (Anselmi et al., 2025; Brancaccio et al., 2025; de Chiusole et al., 2024). Moreover, Stefanutti (2019) proposed the so-called *procedural knowledge space theory* and showed that it can be successfully applied in the field of the assessment of human problem-solving and planning (Stefanutti, de Chiusole, & Brancaccio, 2021; Brancaccio, de Chiusole, & Stefanutti, 2023a). Nevertheless, the application of KST in the field of cognitive psychology is still underexplored.

Along these lines, this paper presents MatriKS, a new computerized tool to measure fluid intelligence, developed using KST and validated with a multi-method approach, including CTT, IRT, and KST. Thus, the novelty of this research is double-faced. First, it applies KST to fluid intelligence assessment, marking a novel use of the theory in this context. Second, it uses CTT, IRT, and KST together to demonstrate that a multi-method approach can enhance the robustness, reliability, and comprehensiveness of test validation. By combining these diverse methodologies, researchers can benefit from the distinct advantages that each method offers, providing a more holistic and effective tool for the assessment of fluid intelligence.

The paper is organized into the following sections. Section “Knowledge structure theory: Construction and validation of cognitive tests” summarizes the key KST-based methods and procedures used in constructing and validating a cognitive test. Section “Automatic rule-based generation of the MatriKS items” provides a detailed description of the principles underlying the creation of the new Raven-like matrices integrated into MatriKS. The multi-method validation analysis is illustrated in Section “Multi-method validation analysis”. This section outlines the administration procedure and participants, followed by a comprehensive presentation of data analyses and results from the perspectives of CTT, IRT, and KST. Considerations on the outcomes of the three approaches are given in Section “Considerations on the outcomes of the three approaches”. The paper concludes with some final remarks.

## Knowledge structure theory: Construction and validation of cognitive tests

The theory of knowledge structures (Doignon & Falmagne, 1985, 1999; Falmagne & Doignon, 2011) is a mathematical approach conceived for the non-numerical assessment of knowledge. Defining a *knowledge domain* *Q* as the set of all problems useful for assessing knowledge in a given field (e.g., mathematics, chemistry, statistics, etc.), the individual’s knowledge is represented by the subset of problems in *Q* they are able to solve. This subset is named *knowledge state*, and it is denoted by *K*. When a population of individuals is considered, it is possible to define the collection $\mathcal {K}$ of all knowledge states existing in that population, named *knowledge structure*. This last contains at least the empty set, representing the individual who knows nothing in the domain, and the full set *Q*, representing the individual who knows everything in the domain.

It is worth noticing that a knowledge structure reflects the precedence relations (e.g., prerequisites) existing among the problems in *Q*. Thus, $\mathcal {K}$ is a subset of the power set $2^Q$ (i.e., the collection of all the subsets that can be obtained from *Q*). In particular, if a problem belongs to a knowledge state, then all of its prerequisites must belong to that knowledge state too.

KST was initially developed as a behavioral theory that makes no assumptions about or descriptions of the cognitive processes or skills underlying problem-solving. Later, the theory was extended to a “competence” level for the assessment of cognitive skills (Doignon, 1994; Düntsch & Gediga, 1995; Falmagne, Koppen, Villano, Doignon, & Johanessen, 1990; Gediga & Düntsch, 2002; Stefanutti & de Chiusole, 2017; Ünlü et al., 2013; Heller, Stefanutti, Anselmi, & Robusto, 2015; Korossy, 1997, 1999). Such extension is known as *competence-based knowledge structure theory* (CbKST; Heller, Unlü, & Albert, 2013b; Heller, Augustin, Hockemeyer, Stefanutti, & Albert, 2013a; Stefanutti & Albert, 2003). Given a set *S* of skills, the *competence state* is the set $C \subseteq S$ of skills mastered by an individual, and the collection $\mathcal {C}$ of all the competence states is the *competence structure*. This last contains at least the empty set, and the full set *S*.

The behavioral and competence levels can be connected by two functions: the *skill map* and the *problem function* (Doignon, 1994; Düntsch & Gediga, 1995; Heller et al., 2013b). The skill map is a triple $(Q, S,\mu )$ where $\mu : Q \rightarrow 2^S$ is a function assigning a nonempty subset of skills in *S* to each item in *Q*. In this way, it connects the performance level to the competence level. Notably, skill maps have two alternative interpretations called *conjunctive* and *disjunctive* (Doignon, 1994). In the former, all the skills in $\mu (q)$ are necessary for solving *q*. In the latter, any skill in $\mu (q)$ is sufficient for solving *q*. Under the conjunctive model (i.e., the approach used in the following), the problem function $p: \mathcal {C} \rightarrow 2^Q$ is defined by

1 $$\begin{aligned} p(C) = \{q \in Q: \mu (q) \subseteq C\}, \end{aligned}$$

for each state $\mathcal {C} \in \mathcal {C}$. The collection of all the *p*(*C*) induced by the states $\mathcal {C} \in \mathcal {C}$ is, indeed, a knowledge structure consisting of knowledge states that are delineated by $\mu$. The knowledge and the competence structures are deterministic models that need to be empirically validated. This can be done by testing the fit of a probabilistic model to some empirical data. The model mostly used for this purpose is the *basic local independence model* (BLIM; Doignon & Falmagne, 1999; Falmagne & Doignon, 1988). The BLIM is a probabilistic model that makes a distinction between the latent knowledge state *K* of an individual and their observable *response pattern* *R* (i.e., the subset of items correctly solved). This distinction between unobservable knowledge states and observable response patterns is posited following the assumption that observed data are noisy and that they might mask the true knowledge states.

Under the BLIM, the relation between *K* and *R* is probabilistic, and it is given by a model in which the marginal probability *P*(*R*) of the response patterns can be computed by

2 $$\begin{aligned} P(R)=\sum _{K \in \mathcal {K}} P(R|K)\pi _K, \end{aligned}$$

where $\pi _K$ is the probability of *K* in the population. Assuming that the responses provided by a student to the items are locally independent, given the true knowledge state of that student, the conditional probability *P*(*R*|*K*) can be computed by

3 $$\begin{aligned} P(R|K)= \left( \prod _{q\in K\setminus R} \beta _q\right) \left( \prod _{q \in K \cap R } (1-\beta _q) \right) \left( \prod _{q \in R \setminus K} \eta _q \right) \left( \prod _{q \in Q \setminus (K \cup R)} (1-\eta _q) \right) , \end{aligned}$$

where $\beta _q \in [0,1)$ is a careless error probability and $\eta _q \in [0,1)$ is a lucky guess probability. The careless error parameter reflects the conditional probability that an individual does not solve item *q* given that *q* belongs to their knowledge state *K*. On the contrary, the lucky guess parameter reflects the conditional probability that an individual solves *q* given that *q* does not belong to their *K*.

The careless error and lucky guess parameters of an item provide a measure of random error in the two directions of, respectively, a false negative and a false positive. Therefore, they also provide an indirect measure of each item’s reliability. In this respect, there is a fundamental assumption behind the BLIM, which is required for having consistent knowledge assessment. According to this assumption, an incorrect response to a problem *q* should be more likely if an individual is not capable of solving *q* (no lucky guess occurred) than if they are (a careless error occurred). Equivalently, a correct response to a problem *q* should be more likely if an individual is capable of solving *q* (no careless error) than if they are not (lucky guess). Formally, the assumption takes on the form of the following inequality: $\beta _q +\eta _q<1$. Items for which the condition turns out to be false should be removed from the test.

It is worth mentioning that two distinct procedures exist for deriving parameter estimates for the BLIM: by maximum-likelihood (ML) via the expectation-maximization (EM) algorithm adapted for KST (Stefanutti & Robusto, 2009; Anselmi, Robusto, Stefanutti, & de Chiusole, 2016; de Chiusole, Stefanutti, Anselmi, & Robusto, 2015) and by minimum discrepancy (Heller & Wickelmaier, 2013). Moreover, the “pks” R package and a MATLAB toolbox provide the functions for applying these two estimation procedures (Brancaccio, de Chiusole, & Wickelmaier, 2024).

The goodness-of-fit of the model to the data can be evaluated using the chi-square statistic and a likelihood ratio test between the estimated and saturated models. If the model fits the data, there is empirical evidence that the items, skills, and structure used to collect the data are plausible in the real world.

Over time, thorough investigations have delved into this model, establishing it as a robust framework for validating knowledge structures. A (non-exhaustive) list of studies on this model is Stefanutti, Heller, Anselmi, and Robusto (2012); de Chiusole, Stefanutti, Anselmi, and Robusto (2013b); Stefanutti and Robusto (2009); Stefanutti, Spoto, and Vidotto (2018); Anselmi, Stefanutti, Chiusole, and Robusto (2017); de Chiusole, Anselmi, Stefanutti, and Robusto (2013a); de Chiusole and Stefanutti (2013); Heller et al. (2015); Heller, Stefanutti, Anselmi, and Robusto (2016).

One of the most important results obtained about the BLIM concerns its local identifiability. If the domain *Q* has a moderate size (e.g., up to about 20 items), a general test of the local identifiability of the BLIM might be performed by using the MATLAB function blimit (Stefanutti et al., 2012). This function tests the local identifiability of the BLIM for arbitrary knowledge structures. If the model is not locally identifiable for a given knowledge structure, the blimit function provides precise diagnostics of the local identifiability of each parameter in the model. When the number of items in *Q* is greater than 20, no analytic procedures can be applied, and the only possibility to test the local identifiability of the model is via an empirical procedure. This last consists of estimating the BLIM with the same data set, each time starting from a different point in the parameter space. If the model is identifiable, the estimated parameter values do not depend on their initial values, and they remain the same (up to round-off error) all the time. If the model is unidentifiable, then the estimated values depend on the initial values and the obtained estimated values will change each time. Thus, the standard deviation of the parameter estimates and the difference between the maximum and the minimum values of the estimates can be analyzed. If parameters are identifiable, their estimates have zero variance (up to round-off error), otherwise they have a nonzero variance. For a similar approach, see, e.g., Stefanutti, de Chiusole, Anselmi, and Spoto (2020) and Stefanutti, de Chiusole, and Brancaccio (2021).

**Table 1:** Taxonomy of the “transformation rules” most commonly used in the literature

Macro-category	Rule	Definition
Configuration elab.	Completion	Identification of the missing portion of an object
Visuospatial	Orientation	Manipulation of the spatial orientation
	Shape	Manipulation of the shapes
	Filling	Manipulation of the shading
	Size	Manipulation of the size
Pre-inference	Object addition	Visual combination of two objects into a whole
	Object subtraction	Visual deletion of the objects across cells
Inference	Conjunction (AND)	The object in the third cell is obtained via the intersection between the objects in the first two cells
	Disjunction (OR)	The object in the third cell is obtained via the union of the elements in the first two cells
	Exclusive disj. (XOR)	The object in the third cell is obtained via the symmetrical difference between the elements in the first two cells
Directional logic	Horizontal	Rules are applied horizontally (across columns)
	Vertical	Rules are applied vertically (across rows)
	Diagonal	Rule are concurrently applied vertically and horizontally

*Note*: The third cell can be the third cell of either a row or a column. The diagonal directional rule can follow the main diagonal of the matrix from the top-left corner to the bottom-right corner (TL-BR directional logic) or the secondary diagonal of the matrix from the bottom-left corner to the top-right corner (BL-TR directional logic)

Having a model available that fits the data and whose item parameter estimates are reliable and identifiable, the assessment of the underlying skills can be performed by assigning to each participant their knowledge and competence states. For each response pattern *R* and each knowledge state $K \in \mathcal {K}$, the conditional probability *P*(*K*|*R*) is computed by an application of the Bayes theorem, with the following formula:

4 $$\begin{aligned} P (K | R) = \frac{P (R|K) \pi _{\mathcal {K}}(K)}{P(R)}, \end{aligned}$$

where *P*(*R*) and *P*(*R*|*K*) are computed, respectively, by Eqs. (2) and (3), and $\pi _{\mathcal {K}}$ are the state probability estimates. For each individual, the *posterior probability distribution* among the states is obtained by computing *P*(*R*|*K*) for all $K \in \mathcal {K}$. Then, the modal state $\hat{K}$ in the posterior probability distribution is taken to be the knowledge state of the individual.

It is worth noting that the same knowledge state can be mapped to different competence states. This happens whenever a 1-to-1 correspondence between knowledge states in $\mathcal {K}$ and competence states in $\mathcal {C}$ does not hold, resulting in a collection of non-singleton equivalence classes among competence states. However, if some dependence among the skills (e.g., in the form of a prerequisite relation) is theoretically plausible, then their introduction in the model might reduce the size of the equivalence classes. In general, when the 1-to-1 correspondence does not hold, the “minimum competence state” can be computed for each knowledge state (Stefanutti & de Chiusole, 2017; de Chiusole, Stefanutti, Anselmi, & Robusto, 2020; Heller, Anselmi, Stefanutti, & Robusto, 2017), which includes only the skills that the individual certainly masters. The minimum competence state $\widehat{C}$ that corresponds to a particular knowledge state $\widehat{K}$ is built by taking the union of the subsets of rules assigned to each item $q \in \widehat{K}$ via the skill map $\mu$, that is:

5 $$\begin{aligned} \widehat{C}=\bigcup _{q \in \widehat{K}} \mu (q). \end{aligned}$$

In this way, for each individual of the sample, the modal knowledge state $\widehat{K}$ and the corresponding modal competence state $\widehat{C}$ are obtained.

## Automatic rule-based generation of the MatriKS items

In this section, the principles that guided the generation of MatriKS items are given and justified in light of the most relevant literature that studied the structure of Raven’s stimuli and the properties that contribute to their difficulty. For an overview, see, e.g., Carpenter, Just, and Shell (1990), DeShon, Chan, and Weissbein (1995), Matzen et al. (2010), and Primi (2001).

A review of the studies cited above allowed for the development of a new and simplified taxonomy that individualizes the so-called “transformation rules” mostly used for creating new Raven-like stimuli. These rules are listed in Table 1 and refer to the following five macro-categories: elaboration of the general configuration, visuospatial transformation, pre-inference reasoning, inference reasoning, and directional logic.

**Fig. 1:**
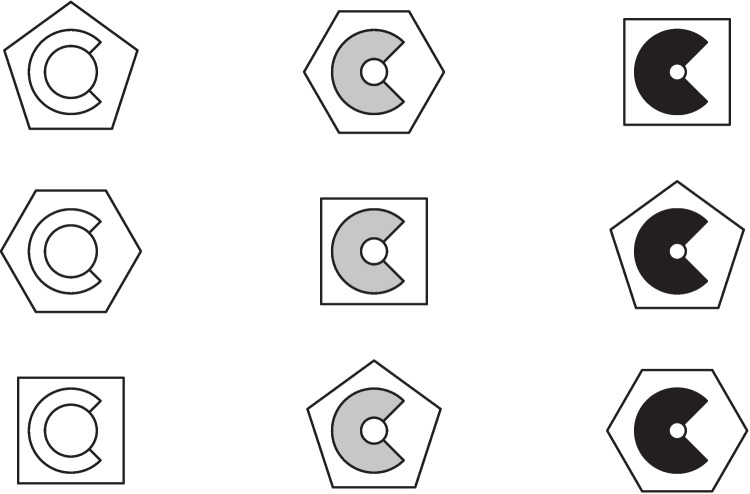
Example of a Raven-like matrix automatically generated manipulating the external shape (diagonal logic), filling (horizontal logic) and size (horizontal logic) transformation rules

Figure 1 shows one Raven-like matrix as an example of the way the transformation rules apply in a matrix.

The manipulated transformation rules are: (i) the external shape, which varies with the so-called top-left to low-right diagonal logic; (ii) the filling of the “pacman”, which varies with a horizontal logic; and (iii) the size of the circle, which varies with a horizontal logic, too.

Individuals aged 4 to 11 were chosen as the target group for the test administration. Thus, all the generative rules belonging to the category *inference reasoning* were excluded. This aligns with Piaget’s stages of cognitive development (Piaget, 1978), which proposes that logical thinking (i.e., the formal operational stage) typically emerges after age 12. All other rules were taken into account when building the MatriKS stimuli.

**Fig. 2:**
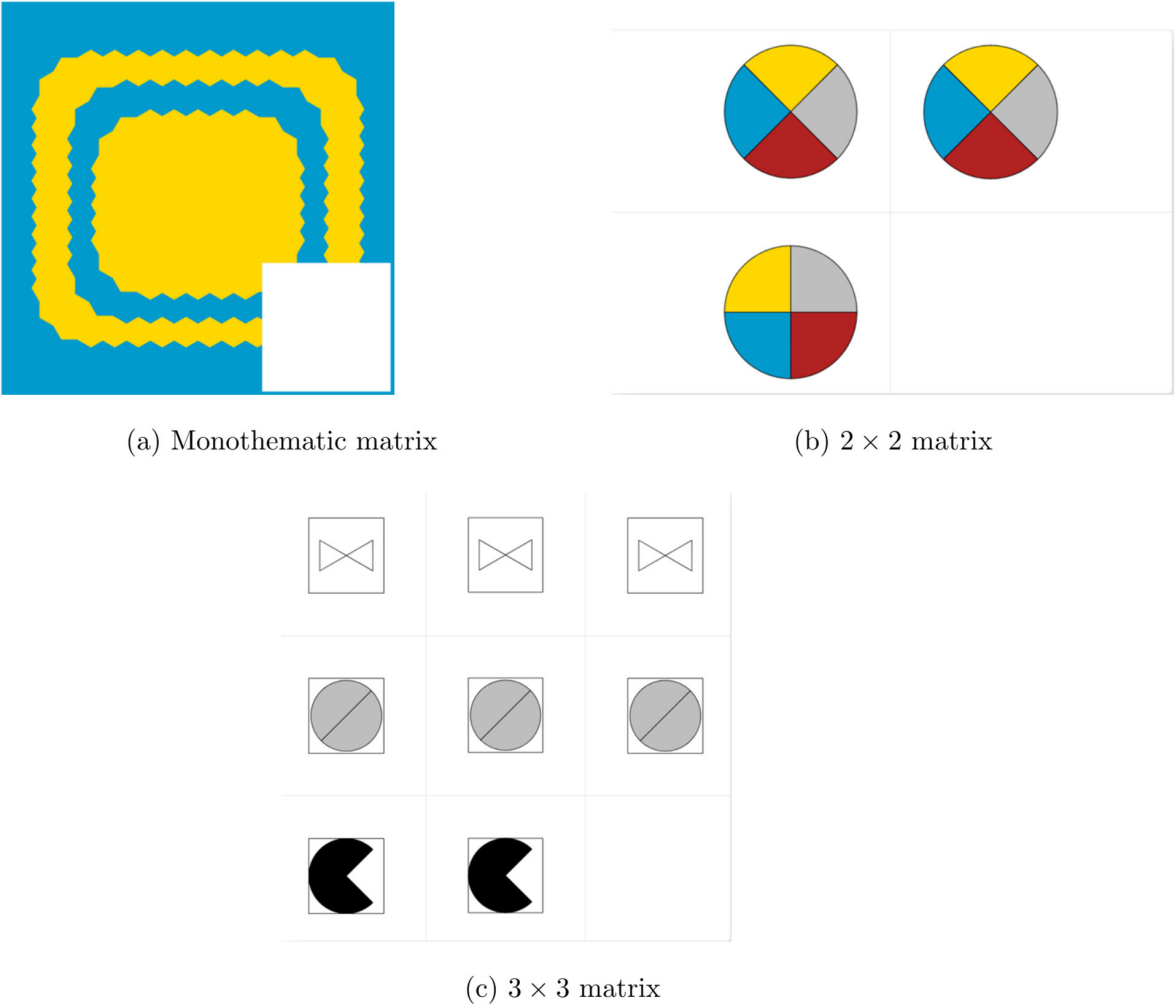
Three examples of Raven-like matrices having different dimensions. Namely a monothematic matrix (Panel **a**), a $2 \times 2$ matrix (Panel **b**), and a $3 \times 3$ matrix (Panel **c**) are depicted

In addition to the transformation rules, Raven-like matrices can be created with different numbers of cells and different types and numbers of response options. Dimensions used in current Raven’s tests are: (a) the so-called “Monothematic” or “jigsaw puzzles” matrices, representing a single figure from which a piece is missing; (b) $2\times 2$ matrices, representing series of four cells, one of which is missing; (c) $3 \times 3$ matrices, representing series of nine cells, one of which is missing. The missing cell is typically at the lower-right corner of the matrix. Figure 2 shows an example for each of the three types of matrices. MatriKS contains all three types of matrices.

**Fig. 3:**
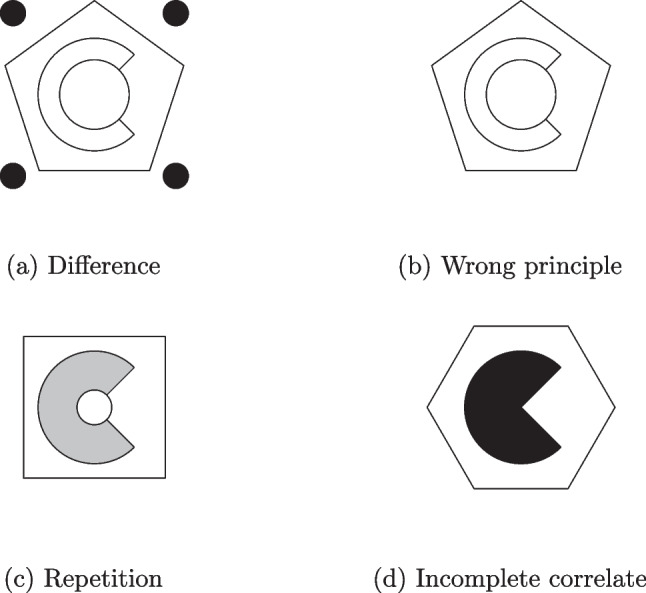
Example of the plausible distractors for the matrix depicted in Fig. 1

The answer to each Raven-like matrix has to be chosen from a list of response options. Only one option is correct, and all the others are wrong. Wrong options are named *distractors*. Studies on common response errors in the Raven’s tests (see, e.g., Kunda, Soulieres, Rozga, & Goel, 2016) led to group distractors into the following four categories: (i) *repetition* refers to an error response corresponding to a cell adjacent to the missing one; (ii) *wrong principle* refers to an error response that applies an incorrect combination of rules, (iii) *difference* refers to an error response characterized by a pop-out effect since it is perceptively different from all the other responses; and (iv) *incomplete correlate* refers to an error response with a missing or modified transformation rule. In MatriKS, the response options were designed to represent each of the four distractor categories. Figure 3 shows one distractor for each of the four types for the matrix depicted in Fig. 1. In MatriKS, response options of the $2\times 2$ and monothematic matrices include one distractor for each macro-category along with the correct response. Thus, an overall of five response options was considered for these matrices. In the $3\times 3$ matrices, two distractors for each category were included in the response options, except for the difference distractor, for which only one distractor was included. This was done intentionally to emphasize the pop-out effect of this distractor. Thus, an overall of eight response options was considered for $3\times 3$ matrices.

In choosing the characteristics of the stimuli for MatriKS, the aim was to create an item pool that is: (1) sufficiently large to leave the possibility to exclude some items (if needed); (2) representative of several combinations of the transformation rules; and (3) composed by items with gradually increasing complexity.

Table 2 displays the features of each of the 40 items of MatriKS. Columns 1 to 4 indicate, respectively, the number of the item, the type of matrix among Monothematic (Mono in the table), $2 \times 2$, or $3 \times 3$, if the matrix is colored or not, and the number of response options. The last column displays the subset of transformation rules manipulated in each matrix.

It is worth noticing that, assuming that an individual is able to solve the matrix if they recognize each of the rules manipulated in it (later on named “rule-to-skill correspondence assumption”), the last column of Table 2 represents what in KST is named skill map $(Q,S,\mu )$, where *Q* is the set of the 40 matrices, *S* is the set of the 11 transformation rules, and $\mu$ is the function assigning to each matrix $q \in Q$ the subset of rules/skills required for solving it.

Table 2 guided the creation of the items. More in detailed, the items were created using an ad-hoc developed R package designed for the automatic rule-based generation of Raven-like matrices (i.e., the matRiks package, Brancaccio, Epifania, & de Chiusole, 2023b; Brancaccio, Epifania, Anselmi, & de Chiusole, 2025b; Epifania, Brancaccio, Anselmi, & de Chiusole, 2026). It generates stimuli based on specified parameters, including: (a) the type of matrix (e.g., 2 $\times$ 2 or 3 $\times$ 3); (b) the objects to be used (e.g., square, circle, etc.); (c) the rules that guide the manipulation (e.g., change in shape, orientation, size, etc.); (d) the direction of the manipulation (e.g., vertical, horizontal, or diagonal). For a comprehensive understanding of the package and its functionalities, readers can refer to the documentation accompanying the matRiks package.

## Multi-method validation analysis

### Participants

The sample was composed of $n = 609$ children (47% female). Data from 40 children with developmental disorders (e.g., ADHD, dyslexia), as well as data from one child with a missing response due to the skipping of an item, were excluded from the analyses. The final sample was composed of $n = 568$ children (47.89% female) aged 4 to 11 (mean = 8.33, *sd*= 2.19). The lower and upper bounds of the age interval are included. This means that the test can be administered from the first day of the 4th year of age to the last day of the 11th year of age.

Data were collected among several Italian primary and middle schools. The informed agreements were given to the parents of the children. Only the children for whom the informed agreement signed by their parents could be retrieved were included in the study. To avoid any form of discrimination, no exclusion criteria were applied at this level, such that every child could participate, given the signed agreement of their parents.

The descriptive statistics of the sample are reported in Table 3, along with the total proportion of correct responses for female and male children according to the different schooling years. The schooling years refer to the number of school years that the children have successfully completed. For instance, 0 schooling years means that children have not yet successfully completed an entire school year (e.g., they are enrolled either in kindergarten or in the first year of elementary school). Three schooling years identify children that had completed three entire school years and are currently enrolled in the fourth class of elementary school. A subsample of children was used to investigate evidence of convergent and discriminant validity (see Section “Assessment tools and administration procedure” for more details). This subsample was composed of 237 children aged 4 to 11 years old ($M = 7.89$, $DS = 2.04$), 51% males ($n = 120$), attending kindergarten ($n = 60$, 25%) or primary school ($n = 177$, 75%). Table 1 of the supplementary material reports the distribution of participants across different school levels. Participants were recruited in two Italian regions: Emilia Romagna ($n = 103$, 43%) and Umbria ($n = 134$, 57%), located in the north and center Italy, respectively.

### Assessment tools and administration procedure

MatriKS was administered to each child individually in a separate classroom by trained researchers. The test was presented on the PsycAssist platform (de Chiusole et al., 2024) available at https://psycassist.fisppa.unipd.it by using a tablet (IoS operating system with a 10.9*in* screen). The administration of MatriKS was carried out with the tablet held horizontally.

The MatriKS test was introduced by a brief animated video (95 s), which presented the test, explained the structure of the stimuli and the correct way to select the response from the response list. After the video presentation, children were presented with four practice items and, then, the test started. There were no time constraints. A response was registered once the child touched one of the response options. If the child struggled to find an answer to an item, the researcher could decide to skip it. No feedback was given about the correctness of the responses provided by the children. The stimuli were presented one at a time in random order.

**Table 2:** The 40 stimuli included in MatriKS. See text for more details

#	Color	Dim.	# Options	$\tau (q)$
1	yes	Mono	5	$\{C\}$
2	yes	Mono	5	$\{C\}$
3	yes	Mono	5	$\{C\}$
4	yes	Mono	5	$\{C,V\}$
5	yes	Mono	5	$\{C,V,H,VH\}$
6	yes	$2\times 2$	5	$\{C,O,V\}$
7	yes	$2\times 2$	5	$\{C,O,V,H,VH,D\}$
8	yes	$2\times 2$	5	$\{C,O,F,V,H,VH\}$
9	yes	$2\times 2$	5	$\{C,F,Sh,V\}$
10	yes	$2\times 2$	5	$\{C,F,Sh,V,H,VH\}$
11	yes	$2\times 2$	5	$\{C,O,Sh,V,H,VH,D\}$
12	yes	$2\times 2$	5	$\{C,O,Sh,V,H,VH\}$
13	no	$2\times 2$	5	$\{C,O,V\}$
14	no	$2\times 2$	5	$\{C,O,V,H,VH,D\}$
15	no	$2\times 2$	5	$\{C,O,Sh,V,H,VH\}$
16	no	$2\times 2$	5	$\{C,F,Sh,V\}$
17	no	$2\times 2$	5	$\{C,F,Sh,V,H,VH\}$
18	no	$2\times 2$	5	$\{C,O,Sh,V,H,VH\}$
19	no	$2\times 2$	5	$\{C,O,Sh,V,H,VH\}$
20	yes	$2\times 2$	5	$\{C,Sh,OA,V\}$
21	yes	$2\times 2$	5	$\{C,Sh,V,H,VH,OS\}$
22	yes	$2\times 2$	5	$\{C,Sh,V,H,VH,OS\}$
23	no	$2\times 2$	5	$\{C,Sh,OS,V\}$
24	no	$2\times 2$	5	$\{C,Sh,V,H,VH,OA\}$
25	no	$2\times 2$	5	$\{C,Sh,V,H,VH,OA\}$
26	yes	$3\times 3$	8	$\{C,F,V\}$
27	yes	$3\times 3$	8	$\{C,S,H\}$
28	yes	$3\times 3$	8	$\{C,F,H\}$
29	yes	$3\times 3$	8	$\{C,Sh,V\}$
30	yes	$3\times 3$	8	$\{C,Sh,S,V,H,VH\}$
31	yes	$3\times 3$	8	$\{C,Sh,V\}$
32	yes	$3\times 3$	8	$\{C,Sh,V\}$
33	no	$3\times 3$	8	$\{C,Sh,V\}$
34	no	$3\times 3$	8	$\{C,Sh,S,V,H,VH\}$
35	no	$3\times 3$	8	$\{C,Sh,V\}$
36	no	$3\times 3$	8	$\{C,F,Sh,V,H,VH\}$
37	no	$3\times 3$	8	$\{C,O,F,H\}$
38	no	$3\times 3$	8	$\{C,O,F,V,H,VH\}$
39	no	$3\times 3$	8	$\{C,O,Sh,S,V,H,VH\}$
40	no	$3\times 3$	8	$\{C,O,F,Sh,V,H,VH\}$

Note: C = Completion; O = Orientation; F = Filling; Sh = Shape; S = Size; V = Vertical Logic; H = Horizontal Logic; VH = Vertical & Horizontal Logic; D = Diagonal Logic; OA = Object Addition; OS = Object Subtraction

**Table 3:** Descriptive statistics of the sample considering schooling years (column 1), gender (column 2), along with the proportion of correct responses (column 6)

Sch. years	Gender	*n*	Age range (*SD*)	Mean age	Prop. correct (*SD*)
0	F	84	4.20 – 7.18	5.60(0.80)	0.47 (0.50)
	M	96	4.19 – 7.36	5.63(0.77)	0.44 (0.50)
1	F	26	7.07 – 8.28	7.68(0.33)	0.70 (0.46)
	M	36	7.12 – 8.12	7.68(0.26)	0.68 (0.47)
2	F	53	7.88 – 10.22	8.68(0.39)	0.77 (0.42)
	M	46	8.14 – 9.37	8.74(0.28)	0.75 (0.43)
3	F	56	9.06 – 10.17	9.67(0.32)	0.82 (0.38)
	M	49	9.16 – 10.21	9.74(0.29)	0.79 (0.40)
4	F	23	10.32 – 11.98	10.94(0.37)	0.83 (0.38)
	M	40	9.30 – 11.41	10.80(0.36)	0.82 (0.38)
5	F	30	11.02 – 12.06	11.59(0.32)	0.86 (0.35)
	M	29	11.12 – 11.90	11.53(0.22)	0.84 (0.37)

To investigate evidence of convergent and discriminant validity, children completed the colored progressive matrices (CPM; Raven, 1954; Belacchi, Scalisi, Cannoni, & Cornoldi, 2008) and the Tower of London test (ToL; Shallice, 1982; Sannio Fancello, Vio, & Ciacchetti, 2021), in addition to MatriKS.

CPM represents one of the most popular measures of non-verbal reasoning abilities for children and elders. It consists of 36 items, increasing in difficulty, organized into three series (i.e., A, AB, and B) with 12 items each. The task consists of identifying the piece that best completes the given matrix, choosing among six alternatives. Series A evaluates the ability to identify similarities (based on shape, dimension, direction, quantity, orientation, figure/background, density criteria), series AB assesses the ability to detect symmetry, and series B measures the conceptual thinking skills (i.e., detection of abstract relations according to operant-deductive logic and their retention in working memory). A score of one is assigned if the person identified the correct option. Otherwise, the score is zero. Thus, the highest total score achievable is 36.

ToL is one of the most popular measures of individuals’ planning abilities across diverse chronological ages. The materials include a wooden base with three pegs of different lengths and three colored beads (red, green, blue). The task consists in moving the beads from a starting configuration, the same for each problem, to a drawn target position, different for each problem, in a given number of moves and respecting three rules (i.e., moving one bead at a time, placing the picked bead into a peg before picking up another one, placing no more than two beads on the middle peg and no more than one bead on the shortest peg). ToL consists of 12 items of increasing difficulty, which require an increasingly greater number of moves to be solved. For this study, a score of one was assigned if the individual reached the target position within the required number of moves at the first attempt. Thus, the highest total score achievable was 12.

The three instruments were administered individually in a quiet room at school, by trained psychologists ($n = 2$) or psychology interns ($n = 9$). Instruments were administered in a counterbalanced order, both for the typology of test (computerized vs traditional, i.e., MatriKS vs CPM and ToL) and the two traditional measures (i.e., CPM and ToL). Table 4 shows the frequencies of these administration orders. Moreover, between the administration of MatriKS and the traditional instruments (or vice versa), there was a delay of three to four weeks.

**Table 4:** Frequencies of tests administration orders

Order	*n*	%
Typology		
Traditional measures – MatriKS	135	57
MatriKS – Traditional measures	102	43
Traditional measures		
CPM – TOL	129	54
TOL – CPM	108	46

### Preliminary analysis

Two items (Items 3 and 7 in Table 2) were removed from the analysis because an inconsistency was detected among the response options. The subsequent analyses are based on an item pool composed of 38 items, which was also the maximum achievable observed score.

The proportions of correct responses for female and male participants in each schooling year are illustrated in Table 3. To test for any significant difference between male and female respondents across schooling years, a generalized linear model (GLM) including the main effects of gender and schooling years, as well as their interaction, was fitted on the proportion of correct responses. The coefficients for each predictor are expressed in log-odds, which can be easily transformed into odds ratios (ORs) by exponentiation. ORs close to 1 indicate little or no association. Values between approximately 1.5 and 3 suggest a moderate effect, while values above 3 indicate a strong effect. ORs below 1 indicate a negative effect, with smaller values (e.g., $OR < 0.67$) representing stronger effects (Bland & Altman, 2000; Chen, Cohen, & Chen, 2010; Davies, Crombie, & Tavakoli, 1998).

To further understand the contribution of each predictor, the $\eta ^2$ effect size measure was computed (Nagelkerke, 1991) with the rsq package (Zhang, 2024) in (R Core Team, 2023). The main effects of schooling ($\beta = 0.47$, SE $= 0.03$, $z = 14.36$, $p <.001$, $\eta ^2 = 0.31$, $OR = 1.60$) and gender ($\beta _M = -0.11$, SE $= 0.04$, $z = -2.54$, $p =.01$, $\eta ^2 = 0.01$, $OR = 0.89$) were significant, while the interaction effect was not ($\beta = -0.02$, SE $= 0.02$, $z = -0.91$, $p =.36$, $\eta ^2 = 0.001$, $OR = 0.98$).

In terms of ORs, schooling was associated with a moderate increase in the likelihood of correct responses ($OR = 1.60$), indicating that each additional year of schooling raised the odds of a correct answer by approximately 60% (Bland & Altman, 2000; Chen et al., 2010). Conversely, the odds ratio for gender ($OR = 0.89$) suggested a negligible and practically irrelevant effect, while the interaction term ($OR = 0.98$) reflected the absence of any meaningful association (Davies et al., 1998).

Overall, the effect size indices based on $\eta ^2$ corroborated the pattern observed for the ORs, confirming that schooling explained the largest proportion of variance in the proportion of correct responses.

### Classical test theory

#### Methods

In this application, *exploratory factor analysis* (EFA) and *confirmatory factor analysis* (CFA; Joreskog, 1969) were used for investigating evidence of the construct validity of the MatriKS score interpretation.

To perform cross-validation of the latent structure of MatriKS using EFA and CFA, the sample was split into two subsamples based on the stratification of gender and schooling year of the whole sample. Stratification by gender and schooling year was employed to preserve the proportional representation of these variables within each sub-sample.

Preliminary steps for conducting EFA and CFA were assessed using Kaiser–Meyer–Olkin (KMO, Kaiser, 1970) measure of sampling adequacy and Bartlett’s test of sphericity. KMO examines the strength of the partial correlation between the items. Values greater than .80 are considered optimal, while values greater than .70 are considered acceptable for running EFA (Kaiser & Rice, 1974). The Bartlett’s test of sphericity is used to test the null hypothesis that the correlation matrix is an identity matrix. To run the factor analysis, the Bartlett’s test needs to be below the nominal level of significance.

EFA was employed to explore and determine the number of latent factors and the pattern of relationships between the indicators and the latent factors. CFA was employed for testing the soundness of the factorial structure and for investigating the reliability of the test.

Among other methods available for determining the dimensionality of a test (e.g., MAP, parallel analysis, eigenvalues), the scree test was employed for determining the number of latent factors to investigate with EFA. Given that MatriKS is a maximum performance test where a correct response is expected and the responses are dichotomically coded as correct or incorrect: (i) the tetrachoric correlations between the items is usually employed as the starting point for EFA, (ii) the weighted least square mean and variance adjusted (WLSMV) estimator is used in EFA, and (iii) the diagonally weighted least squares (DWLS) estimator is used in CFA.

The models investigated with EFA and CFA were considered to have a good fit to the data when the root mean square error of approximation (RMSEA) is equal or lower than .08 (optimal $\le .{06}$, Hu & Bentler, 1999) and the comparative fit index (CFI) and the Tucker–Lewis Index (TLI) are equal to or greater than .95 (Hu & Bentler, 1999). The items with substantial factors loadings on the latent factor(s) (i.e., $a_i \ge .30$) were retained, and, in case of solutions with multiple factors the items with cross-loadings (i.e., items with substantial factor loadings on more than one factor) were discarded. According to Hattie (1985), the following criteria were applied to rule out the over extraction of latent factors and to support the unidimensional model: (i) the first latent factor explains more than 20% of the common variance, (ii) the ratio between the eigenvalues of the first and second latent factors is greater than 3, (iii) the first and second latent factors are strongly correlated (i.e., $>.50$), and (iv) the indicators present substantial factor loadings ($>.30$) on the first latent factor.

Reliability was checked by computing Cronbach’s $\alpha$. A value $\alpha >.85$ suggests an optimal reliability, while an increase of .01 of the $\alpha$ value when an item is removed is sufficient for considering the item as not contributing to the internal consistency of the scale, and it is hence removed (Hattie, 1985).

To investigate the convergent and discriminant validity of MatriKS, the relationships between accuracy scores of MatriKS and those obtained on measures of a similar construct (CPM; convergent validity) and of a different construct (ToL; discriminant validity) were considered. A multilevel modeling framework accounting for the nesting of classrooms within schools represents the most suitable approach to address the dependency among children belonging to the same class and hence sharing the same educational context. However, this approach was not feasible with the current data, as only three schools included more than one classroom. To account for the non-independence of observations, a variable uniquely identifying each classroom within each school was created and modeled as a random intercept in a generalized linear mixed model (GLMM) for binomial responses with a logit link function. The GLMM allowed for the concurrent accounting of both the dependency among observations and the proportional nature of the dependent variable (i.e., the number of correct responses out of the total items composing MatriKS).

The strength of the relationship between the MatriKS score and the scores used for the investigation of convergent (CPM scores) and discriminant (ToL scores) validity is expressed in terms of odds ratio (OR). Since both CPM and ToL scores were standardized prior to model fitting, the reported ORs reflect the multiplicative change in the odds of a correct response on MatriKS associated with a 1 standard deviation increase in the CPM/ToL scores. Moreover, the marginal and conditional $R^2$ were computed to understand the proportion of the MatriKS score explained by the linear combination of the predictors, either by excluding – marginal – or including – conditional – the random effects (Nakagawa & Schielzeth, 2013).

#### Results

EFA and CFA have been applied with the lavaan package (Rosseel, 2012), while Cronbach $\alpha$ has been computed with the psych package (Revelle, 2023) in R (R Core Team, 2023). The lavaan package has also been used to fit a unidimensional CFA model in IRT analysis. The GLMM for the investigation of the convergent and divergent validity of the MatriKS score was fitted with the lme4 package (Bates, Mächler, Bolker, & Walker 2015), while the conditional and marginal $R^2$ for evaluating its predictive power (Nakagawa & Schielzeth, 2013) were computed with the performance package (Lüdecke, Ben-Shachar, Patil, Waggoner, & Makowski, 2021). The first sub-sample ($n = 278$) has been used for running the scree test and EFA. The second sub-sample ($n = 290$) has been used for carrying out the CFA and investigating the reliability of MatriKS. No significant differences were found in the proportion of correct responses between the sub-samples and their stratifications (all $p >.05$).

The Bartlett’s test of sphericity ($\chi ^2=48,795$, $df=703$, $p<.001$) indicated that the tetrachoric correlation matrix was adequate for running both EFA and CFA. Moreover, KMO values ranged between .78 and .95.

The scree plot indicated the extraction of three latent factors. However, while the second and third factors appeared to be closely clustered together and proximate to the elbow, the first latent factor was notably distant from the elbow and the other two latent factors. Parallel analysis suggested the extraction of eight factors, although only the first three observed factors were above the simulated latent factors. Given that: (i) the first latent factor explained more than the 20% of the common variance (i.e., 50%), (ii) the ratio between the eigenvalues of the first two factors was greater than 3 (i.e., 6.52), (iii) the correlation between the first two latent factor was strong (i.e., $r =.60$), and (iv) all items but one (item 18) presented substantial factor loadings on the first latent factor ($a_i \ge 30$), the unidimensional model was preferred. RMSEA and CFI suggested a good fit of the unidimensional model to the data (0.03 and .97, respectively). After removing item 18, the Bartlett’s test of sphericity ($\chi ^2=44,627$, $df=630$, $p<.001$) indicated that the tetrachoric correlation matrix was adequate for running both EFA and CFA. Moreover, KMO values ranged between .88 and .96. Discarding Item 18 led to a slight increase in the proportion of explained common variance (51%), while RMSEA and CFI remained unaltered. All remaining items showed substantial and significant factor loadings, ranging from .46 to .88.

The factor solution found with EFA was further investigated with CFA, where the 1-factor model was fitted to the data, excluding Item 18. CFI, TLI, and RMSEA suggested a good fit of the 1-factor CFA model to the data (.96, .96, and .03, respectively). All items presented substantial and significant factor loadings (reported in Table 5 along with their respective standard errors) on the latent factor. Cronbach $\alpha =.91$ suggested a strong reliability of MatriKS. Moreover, the values of $\alpha$ obtained by leaving one item out did not increase, indicating that no other items (apart from Item 18, which had already been excluded in previous analyses) needed to be removed from the test.

**Table 5:** Standardized factor loadings of the unidimensional model fitted on MatriKS

Item	$a_i$	*SE*	Item	$a_i$	*SE*
1	0.73	0.07	23	0.68	0.06
2	0.84	0.05	24	0.72	0.05
4	0.59	0.08	25	0.58	0.06
5	0.45	0.07	26	0.66	0.05
6	0.74	0.05	27	0.69	0.05
8	0.78	0.04	28	0.71	0.05
9	0.56	0.06	29	0.76	0.06
10	0.65	0.06	30	0.52	0.06
11	0.61	0.06	31	0.75	0.05
12	0.69	0.05	32	0.84	0.03
13	0.56	0.06	33	0.73	0.07
14	0.54	0.07	34	0.43	0.07
15	0.74	0.05	35	0.83	0.06
16	0.53	0.08	36	0.65	0.05
17	0.87	0.04	37	0.73	0.05
19	0.71	0.05	38	0.75	0.05
20	0.66	0.06	39	0.43	0.07
21	0.52	0.08	40	0.63	0.06
22	0.62	0.07			

*Note*: $a_i$: Standardized factor loadings of the items on the latent factor, *SE*: Standard errors of the factor loadings

The GLMM for the investigation of the convergent and discriminant validity of MatriKS showed a satisfactory overall performance, with a marginal $R^2 = 0.76$ and a conditional $R^2 = 0.86$. The high marginal $R^2$ indicates that the fixed effects alone explained a substantial proportion of the MatriKS score variance. The inclusion of the random effect accounting for the educational context variability improved the model’s explanatory power, as suggested by the high value of the conditional $R^2$. The model showed an adequate fit, with residuals approximately centered around zero (median $= 0.37$; $Q_1 = -0.89$; $Q_3 = 1.19$). The extreme residual values might indicate locally less well-fitted cases, but no clear pattern of systematic misfit was observed.

The intercept ($\beta = 0.84$, $SE = 0.06$, $p < 0.001$, OR = 2.31) represents the expected probability of a correct response for average levels of CPM and ToL, which corresponds to approximately a 70% chance of responding correctly.

CPM is a significant positive predictor of MatriKS performance ($\beta = 0.70$, $SE = 0.04$, $p < 0.001$, OR = 2.01), indicating that a one standard deviation increase in CPM roughly doubles the odds of a correct response. Conversely, ToL does not significantly predict performance ($\beta = -0.01$, $SE = 0.03$, $p = 0.87$, OR $\approx$ 1.00), with the odds of a correct response remaining essentially unchanged. This pattern of results supports the convergent validity of MatriKS with CPM and its divergent validity with ToL.

### Item response theory

According to IRT models for dichotomous responses, the probability that person *p* endorses item *i* can be estimated considering the characteristics of both person *p* (as described by the parameter $\theta _p$, indicating the latent trait level of person *p*) and item *i*, as described by different parameters. Different unidimensional IRT models for dichotomous responses exist, which differ according to the number of parameters used for describing the functioning of the items. In the 3-Parameter Logistic Model (3-PL; Lord & Novick, 1968), the probability of a correct response is:

6 $$\begin{aligned} P(x_{pi} = 1|\theta _p, b_i, a_i, c_i) = c_i + (1-c_i) \dfrac{\exp [a_i(\theta _p-b_i)]}{1+\exp [a_i(\theta _p-b_i)]} \end{aligned}$$

where $b_i$ is the location of item *i* on the latent trait $\theta$ (it is also known as difficulty parameter), $a_i$ is the discrimination ability of item *i* (i.e., the ability of the item to distinguish between respondents with different latent trait levels $\theta$), and  is the pseudoguessing parameter of item *i* describing the probability of endorsing the item by chance. Additionally, the discrimination parameters $a_i$ quantify the strength of the relationship between each item *i* and the latent trait $\theta$. Specifically, $a_i$ is a transformation of the factor loadings typically obtained from factor analysis within the CTT framework (see e.g., Cho, 2023).

The item response function in Eq. (6) for each item can be graphically represented with the so-called item characteristics curve (ICC). In this type of graphical representation, the *x*-axis represents the latent trait $\theta$ and the *y*-axis represents the expected probability of observing a correct response, given the person and the item parameters. The logistic function of each item can be used to investigate the changes in the expected probability of a correct response for different levels of the latent trait.

By constraining  in Equation (6) for all the items in a test, the 2-Parameter Logistic model (2-PL; Birnbaum, 1968) is obtained. If the pseudoguessing parameter  and the discrimination parameter $a_i$ in Equation (6) are constrained to 0 and 1 for all items in a test, respectively, the 1-Parameter Logistic (1-PL) model is obtained. The 1-PL model is mathematically equivalent to the Rasch model (Rasch, 1960). Since each IRT model can be derived by imposing or relaxing constraints on the discrimination and pseudoguessing parameters, the 1-PL model is nested within the 2-PL model, which is in turn nested within the 3-PL model. The nested nature of the IRT models allows for the use of the likelihood-ratio test (LRT, see e.g., Embretson & Reise, 2013) for model comparison between two competing models, along with the use of entropy indexes such as the Akaike information criterion (AIC; Akaike, 1973) and the Bayesian information criterion (BIC; Schwarz, 1978).

The choice for the most appropriate IRT model can be based on both a priori theoretical considerations and empirical criteria (e.g., model comparison). In tests where the respondents must produce the correct response (e.g., open-ended question) the probability of guessing the correct response out of luck is virtually null, making the 3-PL less feasible than the 2-PL or the 1-PL models. The choice between these two models depends on the discrimination ability of the items. If all items show the same level of discrimination across different levels of the latent trait, they contribute uniformly to its assessment, making the 1-PL model the most appropriate choice. As such, the specific response pattern of each person *p* does not convey more information than the marginal value obtained for each respondent by summing up their responses across items (i.e., sufficient statistic for the estimation of $\theta$).

When the items of a test present multiple response options, it is reasonable to assume that the respondents may occasionally guess the correct response by luck, making the 3-PL model the most appropriate one. Adding up on the effect of the discrimination parameters, the presence of the pseudoguessing parameter implies that correct responses to different items do not convey the same information with respect to the latent trait. As in the 2-PL model, two respondents with the same observed score but different response patterns may obtain different estimates of $\theta$.

IRT models offer the possibility of evaluating the measurement precision of each item with respect to different latent trait levels by means of the item information function (IIF), which can be computed as:

7 $$\begin{aligned} \text {IIF}_{i}(\theta ) = \dfrac{a_i^2[P(\theta )-c_i]^2}{(1-c_i)^2 P(\theta )Q(\theta )}. \end{aligned}$$

where $P(\theta )$ is the probability of observing a correct response according to Eq. (6) and $Q(\theta ) = 1 - P(\theta )$. Depending on the specific IRT model that is fitted, the $a_i$ and the  may be constrained to be 1 or 0, respectively.

From Eq. (7), it appears clear that the IIF varies as a function of the $b_i$, $a_i$, and  parameters. Specifically: (i) the item is more informative (i.e., higher IIF) for $\theta$ levels that are close to the item location $b_i$ than for $\theta$ levels far from $b_i$; (ii) the discrimination parameter $a_i$ plays a central role in determining the IIF, such that the higher the discrimination ability of the item, the higher its IIF, and (iii) items with positive  generally are less informative (i.e., lower IIF), and the higher the pseudoguessing parameter, the lower the information. In the absence of pseudoguessing (i.e.,  ), the maximum of the IIF is obtained for $\theta = b_i$. The pseudoguessing parameter highly affects the precision with which the items measure the latent trait.

The test information function (TIF) is a measure of the overall information of a test with respect to different levels of the latent trait, and it is obtained as the sum of the IIFs across items. The shape (i.e., the regions of the latent trait for which the test is informative) as well as the height (i.e., the amount of information of the test for different regions of the latent trait) strongly depend on the locations of the items along the latent trait, and, if considered, on the discrimination and the pseudoguessing parameters.

The standard error of estimation (SE) describes the measurement precision of the test with respect to different latent trait levels:

8 $$\begin{aligned} \text {SE}(\theta ) = \sqrt{1/\text {TIF}(\theta )}. \end{aligned}$$

The SE and TIF are inversely related: the higher the information for specific latent trait levels, the lower the SE and the higher the measurement precision for those specific levels. Among the factors influencing the SE, the quality of the items in a test – together with the length of the test and the degree of match between item difficulties and the latent trait levels of the respondents – plays a key role. Specifically, higher TIFs and lower SEs are typically observed in longer tests that include highly discriminating items with a low probability of guessing (see, e.g., Hambleton, Swaminathan, & Rogers, 1991).

Three main assumptions need to be met in order to obtain meaningful estimates from IRT models: (i) the items must comply to a monotonic function (i.e., the number of correct responses to each item monotonically increases as the level of the latent trait increases), (ii) the latent trait on which the person and item characteristics lie is unidimensional, and (iii) once the effect of the latent variable is taken into account, the correlation between the observed responses of any pairs of items must be close to 0 (i.e., local independence).

The next section illustrates the methods used for testing these assumptions, along with the criteria that have been considered for validating MatriKS within an IRT-framework.

#### Method

To test the monotonicity assumption of the items, Mokken (1971) suggested the so-called *H* coefficient. Values of the *H* coefficient equal to or lower than .30 suggest that the item does not comply with the monotonic function and should hence be discarded.

In Section “Results”, the CFA selected the 1-factor model, supporting the unidimensionality of the latent variable underlying MatriKS. This may suggest that MatriKS could be unidimensional also when analyzed in IRT framework. However, the CTT validation highlighted the variability between the factor loadings of the items, suggesting that the assumption of the equal discrimination of the items made in the 1-PL model cannot hold. Moreover, since MatriKS is a multiple-choice test where the correct response is chosen among a response list and it may be guessed, the 3-PL model might be the most appropriate for the IRT validation of MatriKS.

**Table 6:** Model comparison between the IRT models fitted on the starting item pool of 34 items

Model	AIC	BIC	logLike	$\chi ^2$	$\Delta$ df	p
1-PL	16720.42	16868.05	$-8326.208$	–	–	–
2-PL	16386.83	16682.09	$-8125.413$	401.589	34	$<.001$
3-PL	16316.51	16759.41	$-8056.257$	138.312	34	$<.001$

Note: $\Delta$ df: Difference between the degrees of freedom of the two competing models

In addition to these considerations, the best-fitting IRT model is chosen by model comparison, considering the Akaike information criterion (AIC), the Bayesian information criterion (BIC), and the likelihood ratio (LR) test. Although model comparison allows for choosing the model that best explains the data among the competing models, it does not provide any information concerning the absolute fit (i.e., how well the model approximates the observed data) of the model (see, e.g., Brown, Templin, & Cohen, 2015). The absolute fit of the IRT model chosen via model comparison is assessed with the M2 statistic and its associated *p* value (Cai & Hansen, 2013; Maydeu-Olivares & Joe, 2005) with $\alpha =.05$. RMSEA and SRMR derived from the M2 statistic are reported as descriptive indices of global fit. In this light, RMSEA values $<.04$ and SRMR $<.08$ denote an acceptable fit (Maydeu-Olivares, 2013).

The item fit is considered as well by using the Orlando–Thissen $S-X^2$ statistic (Orlando & Thissen, 2000, 2003) with $\alpha =.05$. Moreover, RMSEA values based on the $S-X^2$ statistic were considered as well for evaluating item fit, with values lower than .05 indicating an optimal fit and values lower than .08 indicating an acceptable fit.

Local independence is evaluated by correlating the standardized residuals of each pair of items and interpreting the results according to the *Q*3 statistics (Yen, 1984). Values of *Q*3 greater than .20 suggest local dependence between a pair of items (Davidson, Keating, & Eyres, 2004), although other critical values could be considered (see, e.g., González-de Paz et al., 2015; La Porta et al., 2011).

If the 3-PL model results as the best fitting one among the competing IRT models, the pseudoguessing parameter  is considered as well for evaluating the quality of the selected items. Different guidelines exist for evaluating the quality of the items based on the pseudoguessing parameter. For instance, Birnbaum (1968) suggests that items with  (where *k* is the number of response options for item *i*) should be discarded because it means that the correct response might be guessed above chance. The number of response options in MatriKS is five or eight. Thus, a conservative threshold is $1/5 =.20$. This value is also in accordance with Baker and Kim (2004) who suggested a threshold value around .20. For these reasons, the items presenting with  are discarded.

Once the best IRT model is chosen via model comparison at the first step, the validation procedure is repeated until the global fit of the model is reached, no misfitting items are retained, no local dependence is found, and, if the 3-PL model is the chosen one, no items with pseudoguessing parameters greater than .20 are retained.

The IRT validation has been carried out with the mirt package (Chalmers, 2012) in R (R Core Team, 2023), while the graphical representations have been obtained with the ggplot2 package (Wickham, 2016).

#### Results

After checking for monotonicity, four items (Items 5, 18, 34, and 39) presented an *H* coefficient below the threshold and were hence removed from the item pool. The three IRT models were hence fitted on a starting item pool composed of 34 items. The results of model comparison are reported in Table 6. The 3-PL model resulted as the best-fitting model. Also, from a theoretical perspective, the 3-PL model is the most appropriate given that the respondents might guess the correct response out of luck from the response options presented with each item. The IRT validation of MatriKS is hence based on the 3-PL model.

Table 7 illustrates the process undertaken for the IRT validation of MatriKS with the 3-PL model. The first row (denoted as “Start”) refers to the 3-PL model fitted on the starting item pool composed of 34 items, while the last row refers to the final item section found after refining the test according to the following criteria (further described in Section “Method”): (i) global fit of the model according to the *p* value of the *M*2 statistics, the RMSEA, and the SRMSR, (ii) item fit according to the *p* value associated to $\chi ^2$ statistics of each item and its associated RMSEA, (iii) lack of local dependence with other items, and (iv) pseudoguessing parameter below .20.

**Table 7:** Item response theory validation of MatriKS with the 3-PL model

Step	M2	df	*p*	RMSEA	SRMSR	Item Misfit	LD	
Start	678.27	493	$<.001$	0.03	0.05	9, 19	28, 37, 36, 40	1, 2, 4, 8, 12, 25, 28, 36, 37, 40
2	214.36	187	0.08	0.02	0.04			27
3	193.42	168	0.09	0.02	0.04	31, 35, 38		
4	140.79	117	0.07	0.02	0.05	11		
5	124.52	102	0.06	0.02	0.05	32		
6	106.93	88	0.08	0.02	0.05	16,20		
End	74.05	63	0.16	0.02	0.04			

Note: LD: Local dependence measured with the *Q*3 statistics,  : guessing parameter greater than .20

**Fig. 4:**
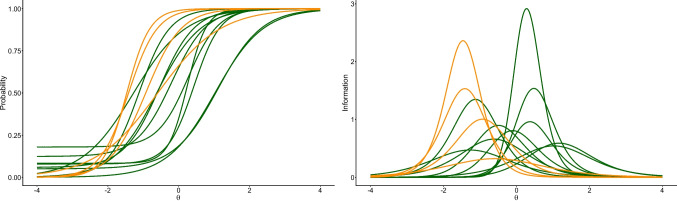
Item characteristics curves (*left panel*) and item information functions (*right panel*) of the final item pool of MatriKS. The *green lines* represent the four-cell items, the *orange lines* represent the nine-cell items

Despite being the model best fitting the data according to model comparison (see Table 6), the 3-PL did not fit the data according to the *M*2 statistics ($p<.001$), although the other fit indexes (RMSEA and SRMSR) suggested an acceptable fit to the data. The lack of global fit might be due to different reasons, among which are misfitting items (Items 9 and 19), items with local dependence (Items 28, 37, 36, 40), and items with guessing parameters above .20. All items that showed local dependence had a pseudoguessing parameter greater than .20. Taken together, this pattern of results might suggest the contribution of more than one dimension in guiding the correct response process. All monothematic puzzles (Items 1, 2, 4) showed a pseudoguessing parameter greater than .20, suggesting that their correct solution can be found out of luck and not only because the person knows the correct response.

All these items have been removed, and the 3-PL has been fitted again on the new set of items. Despite the global fit of the model being barely acceptable at the second step (i.e., *p* value $=.08$), items with either pseudoguessing parameters above the threshold (Item 27, second step) or misfitting items (Steps 3 to 6) were found, and hence the procedure was repeated. After removing the local-dependent items from the starting pool, no further local dependencies were found.

The final model (End in the table) showed an optimal fit after removing 20 items, hence the final version of MatriKS validated within an IRT framework with the 3-PL model is composed of 14 items. The ICCs of the final version of MatriKS are illustrated in the left panel of Fig. 4, while the item parameters along with the item fit measures are illustrated in Table 8.

The two rightmost lines in the left panel of Fig. 4 illustrate the ICCs of two 4-cell items, specifically of items 21 and 22. According to Table 2, these items are supposed to be the most complex ones in terms of mastered rules (i.e., pre-logic rules), and they are the most difficult items according to the 3-PL. Only the four-cell items present a lucky guess parameter greater than 0 (Items 10, 14, 15, 17, 21, 24), while the nine-cell items do not. Item 14 presents the highest pseudoguessing parameter, but it is in line with the chance probability of guessing the correct response out of luck given the number of response options.

From the IIFs represented in the right panel of Fig. 4, it appears that there is a clear separation between the information provided by the nine-cell items (mostly located on the left side of the latent trait) and that provided by the four-cell items (mostly located on the right side of the latent trait). In other words, nine-cell items appear to be more informative for medium to low levels of the latent trait, while four-cell items appear to be more informative for medium to high levels.

**Fig. 5:**
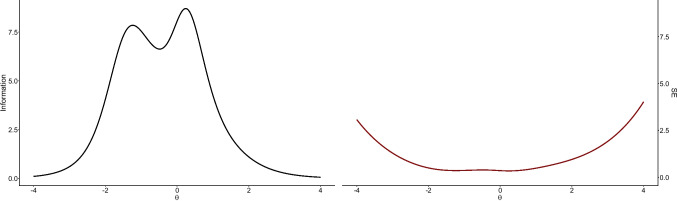
Test information function (*left panel*) and standard error of measurement (*right panel*) of MatriKS

The test information function (TIF), along with the standard error of measurement, is reported in Fig. 5.

Consistently with what is observed on the IIFs in the right panel of Fig. 4, MatriKS is highly informative for latent trait levels around the mean 0, although the information on this specific latent trait level is slightly lower. Nonetheless, by combining the information of the TIF with that of the SEM concerning the precision of measurement, it can be said that MatriKS provides reliable assessment for latent trait levels ranging between $-2$ and $+2$.

The IRT validation of MatriKS pursued with the application of the 3-PL model led to the elimination of 20 items (plus 4 items eliminated because of a lack of monotonicity), resulting in a test composed of 14 items. All monothematic puzzles were discarded; the final test does not present any item that relies solely on the completion rule. Nonetheless, all other rules and directional logics are still represented in the final version of the test.

**Table 8:** Item parameters and fit measures of the final version of MatriKS

Item	$b_i$	$a_i$		S-$X^2$	df	*p* value	RMSEA
6	$-1.12$	2.32	0.00	2.36	6	0.88	0.00
10	−0.20	2.02	0.12	13.24	9	0.15	0.03
13	$-0.62$	1.62	0.00	8.66	9	0.47	0.00
14	0.27	2.33	0.18	14.41	10	0.15	0.03
15	$-0.54$	2.01	0.06	7.09	8	0.53	0.00
17	0.24	3.70	0.08	3.45	8	0.90	0.00
21	1.11	1.61	0.05	10.82	8	0.21	0.02
22	1.02	1.47	0.00	4.77	7	0.69	0.00
23	−1.27	1.37	0.00	14.46	9	0.11	0.03
24	0.43	2.68	0.08	6.56	8	0.58	0.00
26	−0.93	2.00	0.00	12.82	8	0.12	0.03
29	−1.41	2.48	0.00	4.78	6	0.57	0.00
30	$-0.59$	1.13	0.00	6.19	9	0.72	0.00
33	−1.46	3.07	0.00	2.60	5	0.76	0.00

*Note:* Items from 6 to 24 are $2 \times 2$ matrices, items from 26 to 33 are $3 \times 3$ matrices

### Knowledge structure theory

#### Methods

The skill map $(Q,S,\mu )$ depicted in the last column of Table 2 was used for constructing the knowledge structure of the test under the conjunctive model. A knowledge structure $\mathcal {K}$ of 117 states was obtained. It is important to note that the skill map was built under the rule-to-skill correspondence assumption. This assumption states that each rule used to build a matrix directly matches the skill needed to recognize it. This implies that the cognitive requirements for solving a matrix can be fully described by the set of rules applied in its construction. This assumption might be too strong since, beyond the recognition of all the transformation rules manipulated in generating the matrix, other more intuitive mechanisms could lead to the correct response of an individual. Thus, the empirical test is a crucial phase for understanding if the rule-to-skill correspondence assumption can be retained.

Some “dependencies” among the 11 transformation rules considered for building the 38 matrices of the test were theoretically plausible. Defining dependencies among rules has the advantage of reducing the size of the equivalence classes on the competence states. In particular, the following assumptions were considered: (i) the Completion rule represents a minimum requirement (i.e., a prerequisite) for mastering all the other rules, as it is assigned to all matrices in the test. This assumption is justified by the fact that identifying the missing element in a pattern is a necessary cognitive step to find any transformation rule in a matrix; (ii) Vertical Logic rule (*V*) is a prerequisite for Vertical & Horizontal Logic rule (*VH*); (iii) Horizontal Logic rule (*H*) is also a prerequisite for *VH*; (iv) *VH* is a prerequisite of Diagonal Logic (*D*). Assumptions (ii) and (iii) are very plausible in practice, since *VH* rule explicitly combines *V* and *H*, applying them to different objects appearing in the matrix. In this sense, *VH* rule does not introduce a completely new operation, but rather requires the coordinated application of the two underlying rules. Also, assumption (iv) is very plausible in practice, since it requires that *VH* Logic be applied to the same object. Based on the assumptions described above, a competence structure consisting of 385 states was derived. Since a 1-to-1 correspondence between $K \in \mathcal {K}$ and $\mathcal {C} \in \mathcal {C}$ does not hold, an equivalence relation among the competence states exists. Specifically, all competence states delineating the same knowledge state form an equivalence class.

The degree of uncertainty within each equivalence class can be characterized through a set of statistical indices, each capturing a different aspect of the variability among competence states in the same class. Specifically, for each equivalence class [*C*], with $\mathcal {C} \in \mathcal {C}$, the following indices were obtained: (1) the number of skills each of which belongs to all states in [*C*], computed as $|\bigcap [C]|$; (2) the number of skills each of which belong to no states in [*C*], computed as $|S \setminus \bigcup [C]|$; (3) the sum of the two numbers obtained in points (1) and (2), named “certain”; (4) the difference between the number of skills in *S* and the number obtained in point (3), named “uncertain”; (5) the mean canonical distance (i.e., given any two sets *X* and *Y* of *S*, the canonical distance is $|X \Delta Y|$, where $\Delta$ is the symmetric difference between sets *X* and *Y*) is computed across all pairs of competence states belonging to the same class; and (6) the probability $\pi _{|[C]|}$, computed as the sum of the probabilities of all knowledge states delineated within equivalence classes having the same cardinality |[*C*]|.

The indices computed in points (1) and (2) can be interpreted as the number of skills that an individual masters and does not master for sure, respectively. The third index quantifies the number of skills whose classification, as either mastered or not, is certain. The fourth index can be regarded as the number of skills for which there is uncertainty about whether they have been mastered. The fifth index is a measure of dissimilarity across the states in [*C*]. The last index reflects the expected proportion of individuals whose competence state falls in an equivalence class of a given cardinality.

These indices were computed for the structures considered in this application to evaluate the degree of uncertainty in the competence states assigned to individuals. The BLIM was applied to the collected data, and its parameters were estimated by ML (Stefanutti & Robusto, 2009). The absolute goodness-of-fit of the model was assessed using the Pearson chi-square statistic. It is known that the approximation to the asymptotic distribution of the chi-square statistic lacks accuracy for large and sparse data matrices. Thus, a parametric bootstrap procedure over 1000 replications was used to compute the *p* value of the chi-square.

The identifiability of the BLIM parameters was checked by applying the empirical procedure described in Section “Knowledge structure theory: Construction and validation of cognitive tests”. In particular, the estimation of the BLIM’s parameters was repeated 1000 times, each time starting from a different point in the interval (0, .50]. The estimates obtained in the single repetition were retained only if the termination criterion of $10^{-3}$ was reached by the EM algorithm. Otherwise, the obtained estimates were discarded, and the algorithm started again. Then, the standard deviation of the parameter estimates and the difference between the maximum and the minimum values of the estimates were computed. If parameters are identifiable, then the standard deviation of the estimates is expected to be very close to zero.

Item reliability was tested by checking if condition $\beta _q+\eta _q<1$ holds for all items. Items identified as unreliable were removed from the knowledge domain, the skill map, and the data. A new knowledge structure was built and the BLIM was applied again to the data. This procedure was reiterated until all the items turned out to be reliable.

Having available a model that fits the data and whose item parameter estimates are reliable, the knowledge state $\widehat{K}$ and the corresponding minimum competence state $\widehat{C}$ were estimated for each participant by Eq. (4) and (5), respectively. On the basis of these estimates, two indices were computed. The first one is the *average proportion of rules mastered* by individuals in the general population. It was computed by:

9 $$\begin{aligned} p=\frac{\sum _{i=1}^N |\widehat{C}_i|}{|S| N}, \end{aligned}$$

where *N* is the sample size and $\widehat{C}_{i}$ is the minimum competence state corresponding to the modal knowledge state estimated for an individual *i* (given a set *X*, the notation |*X*| stands for its cardinality). This index was computed separately for the six different schooling years 0 to 5.

**Table 9:** Statistical indexes for the equivalence classes of competence states delineating the same knowledge state

|[*C*]|	F	$$|\bigcap [C]|$$	$$| S \setminus \bigcap [C]|$$	Certain	Uncertain	$$|X \Delta Y|$$	$$\pi _{[C]}$$
1	57	7.65	3.35	11	0	0	.79
2	24	6.50	3.50	10	1	1.00	.08
3	8	4.50	4.50	9	2	1.33	.03
4	14	5.79	3.21	9	2	1.33	.01
8	10	4.30	3.70	8	3	1.71	.06
12	2	2.50	4.50	7	4	2.06	.00
16	1	3.00	4.00	7	4	2.13	.00
80	1	1.00	3.00	4	7	3.32	.02

The second index is the *rule probability* $P_s$, that, for each rule $s \in S$, was computed as

10 $$\begin{aligned} P_s=\sum _{C \in \mathcal {C}_{s}} \pi _{p(C)}, \end{aligned}$$

where $\mathcal {C}_{s}$ is the collection of all the minimum competence states containing rule *s*, and *p*(*C*) is the knowledge state obtained by applying the problem function in Eq. (1). In general, $P_s$ is the marginal probability that an individual in the population masters rule *s*. Because it is defined from the set of minimal competence states containing rule *s*, it is a lower bound of this probability. This index was estimated by maximum likelihood separately for each rule and each of the six schooling years 0 to 5. To this aim, an average posterior probability distribution $\pi$ was estimated for each schooling year by simply computing the mean of the estimated posterior probability distributions of the individuals belonging to the same schooling year. The estimated value $\hat{\pi }$ replaced that of $\pi$ in Eq. (10), to obtain a maximum likelihood estimation of $P_s$. It is expected is that both *p* and $P_s$ are monotonically increasing in the schooling years.

#### Results

For running all the data analyses, the MATLAB toolbox “kst” (Brancaccio et al., 2024) was used. It is available from the MATLAB file exchange at https://it.mathworks.com/matlabcentral/fileexchange/157751-kst-toolbox.

The knowledge structure $\mathcal {K}$ obtained with the skill map depicted in the last column of Table 2 is characterized by 117 knowledge states. Since the competence structure comprised 385 states, a 1-to-1 correspondence does not hold. The degree of uncertainty of the competence states delineated by the same knowledge state was evaluated by the six statistical indexes introduced in Section “Knowledge structure theory” (Table 9). The analysis of the equivalence classes revealed that only four of them exhibit significant uncertainty, as shown in the last three rows of the table. Specifically, the uncertainty in assessing individuals whose competence state falls within any of these four classes amounts to four or more skills. Nevertheless, these individuals represent only 2% of the population (based on the sum of the last three probabilities in the last column of Table 9). On the other hand, the 79% of the population falls within classes where there is no uncertainty concerning skills. These results suggest that the overall amount of information for the skill assessment in MatriKS is satisfactory.

The BLIM based on $\mathcal {K}$ applied to the data exhibited a non-significant bootstrap *p*-value $=.15$. Thus, the model fitted the data well. Moreover, all items turned out to be reliable.

Concerning the identifiability of the model, the standard deviation of both the $\beta _q$ and $\eta _q$ estimates turned out to be very close to zero. The highest difference between the maximum and the minimum estimates was $1.6 \times 10^{-3}$ for the $\beta _q$ and $2.7 \times 10^{-3}$ for the $\eta _q$. These values support the model’s identifiability.

**Fig. 6:**
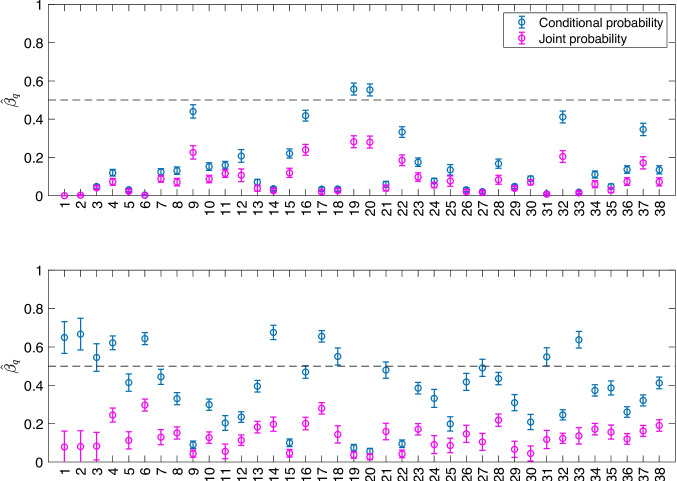
The BLIM’s item error parameters estimated on the data. The *top panel* refers to $\beta _q$ parameters, whereas the *bottom panel* refers to $\eta _q$. In each panel, the conditional (*blue*) and the joint (*magenta*) probability is displayed. *Dashed lines* are for reference

It is worth mentioning that $\beta _q$ and $\eta _q$ estimates are conditional probabilities and, as such, they are affected by the frequency that the item responses have in the sample (i.e., the item probability). In particular, if the frequency of observing a correct response to an item is very high (item probability close to 1), the conditional probability $\eta _q$ can be overestimated. Similarly, if the frequency of observing a correct response is very small (item probability close to 0), the conditional probability $\beta _q$ can be overestimated. This is an issue of ML estimation that arises when there is not enough information in the sample for assuring an unbiased estimate of the parameter (see, e.g., Stefanutti et al., 2020; de Chiusole et al., 2013b). A way to overcome this problem is to estimate joint probabilities instead of conditional probabilities. For each item *q*, the joint probability of $\beta _q$ was computed by multiplying $\beta _q$ times $\pi _q$, whereas the joint probability of $\eta _q$ was computed by multiplying $\eta _q$ times $1-\pi _q$.

Figure 6 shows the $\beta _q$ (top panel) and $\eta _q$ (bottom panel) parameter estimates obtained by applying the BLIM with $\mathcal {K}$. In both panels, the conditional (blue circles) and the joint (magenta circles) probabilities are displayed. Labels of the *x*-axis refer to the ID number of the matrices, as defined in Table 2. The dashed line is for reference. The conditional careless error probabilities are, in general, quite small, whereas those of the lucky guesses are quite high. High values for the lucky-guess estimates can be due to several reasons. One of them is the use of multiple-choice items, which may increase the chance of guessing correctly. Another possible explanation may lie in the high probabilities of correctly solving the items, as mentioned above. This last was indeed the case, because the average item probability is .63, with a minimum value of .50 and a maximum of .88. The joint values of both parameters were quite small: the average value of the joint $\beta _q$ was .08 ($sd=.08$) and that of $\eta _q$ was .13 ($sd=.07$). Overall, these results indicate that the knowledge structure of MatriKS is plausible and can be used to assess the skills related to fluid intelligence.

The performance of individuals in the general population is described here by means of the average proportion of rules mastered *p* and the rules’ probability $P_s$ indexes. For each schooling year, Table 10 shows the average proportion of rules mastered *p*, and the corresponding standard deviation, estimated for the general population. The average value of this index increases monotonically across the schooling years, while its standard deviation decreases. This suggests that fluid intelligence in the general population improves as children’s cognitive systems develop, and that individual variability decreases over time. These results are consistent with the relevant literature (see, e.g., Horn, 2007; Schroeders, Schipolowski, & Wilhelm, 2015). Additionally, the average proportion of rules mastered increases rapidly until year 3 of schooling, and then approaches 95%. This finding indicates that MatriKS is an adequate tool for assessing fluid intelligence in individuals aged 4 to 11 within the general population.

**Table 10:** Average values and standard deviations of the proportion of rule mastered index estimated in the general population for schooling years 0 to 5

Schooling year	Mean	Sd	Schooling year	Mean	Sd
0	0.32	0.33	3	0.91	0.19
1	0.78	0.26	4	0.95	0.12
2	0.86	0.23	5	0.95	0.14

**Fig. 7:**
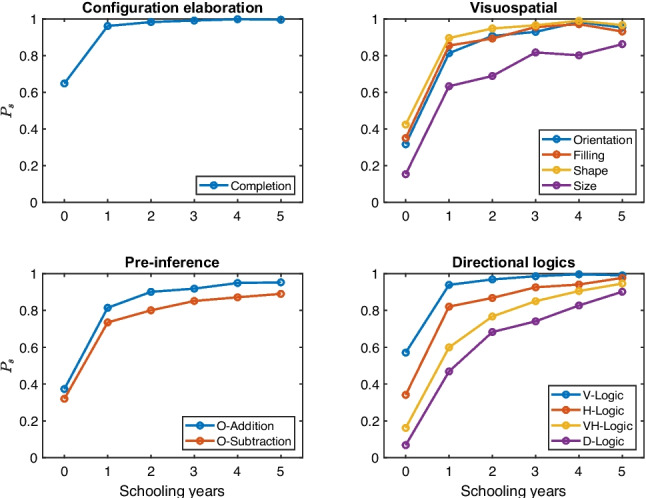
Rule mastering proportion as a function of the schooling years. The *top panels* refer to configuration elaboration (*on the left*) and visuospatial (*on the right*) transformation rules. In contrast, the *bottom panels* refer to pre-inference (*on the left*) and directional logic (*on the right*) rules

Figure 7 shows the results for the rule probability $P_s$ index, computed for all 11 transformation rules. The top panels refer to configuration elaboration (on the left) and visuospatial (on the right) transformation rules. The bottom panels refer to pre-inference (on the left) and directional logic (on the right) rules. The monotonic increase in schooling years was observed for almost all rules. There were very few exceptions (e.g., for rule Filling at schooling year 5), but by a small amount (the biggest drop was .08). In contrast, standard deviations of this index were monotonically decreasing in the schooling year, with very few exceptions (see Table 11). This means that, in the general population, as children’s cognitive systems develop, the variability of their fluid intelligence decreases. Configuration elaboration transformation rule (top left panel) was the easiest rule. Indeed, its probability was higher than 60% also in preschool children. Among visuospatial rules (top right panel), Shape appeared to be the easiest one, whereas size appeared to be the most difficult one (less than 20% of preschool children mastered this rule).

Interestingly, objective addition and subtraction pre-inference rules (bottom left panel) showed very similar probabilities of being mastered at all schooling years, with objective addition rule being slightly easier than the objective subtraction rule.

Finally, the bottom right panel of the figure displays the results of the four directional logic transformation rules. Among these rules, it seems that a dominance relation exists, with the following order (from the easiest to the most difficult): vertical only, horizontal only, vertical and horizontal, diagonal. This last, in particular, is the most difficult rule, with a probability less than 10% to be mastered by preschool children.

**Table 11:** Standard deviations of rule probability index estimated in the general population for schooling years 0 to 5

Schooling year	C	O	F	Sh	S	V	H	VH	D	OA	OS
0	0.48	0.47	0.48	0.49	0.36	0.49	0.47	0.37	0.25	0.48	0.47
1	0.19	0.39	0.35	0.31	0.48	0.24	0.38	0.49	0.50	0.39	0.44
2	0.13	0.29	0.31	0.22	0.46	0.17	0.34	0.42	0.47	0.30	0.40
3	0.09	0.26	0.21	0.18	0.39	0.12	0.26	0.36	0.44	0.27	0.36
4	0.04	0.13	0.17	0.10	0.40	0.06	0.24	0.29	0.38	0.22	0.33
5	0.07	0.21	0.25	0.18	0.34	0.09	0.15	0.23	0.30	0.21	0.31

Note: C = Completion; O = Orientation; F = Filling; Sh = Shape; S = Size; V= Vertical Logic; H = Horizontal Logic; VH = Vertical & Horizontal Logic; D = Diagonal Logic; OA = Object Addition; OS = Object Subtraction

## Considerations on the outcomes of the three approaches

For the multi-method validation of MatriKS, a model was validated within each of the three frameworks CTT, IRT, and KST. The three approaches led to a very different selection of items. Specifically, IRT validation led to the deletion of 24 items from the original item pool, CTT validation discarded one item, while KST validation allowed for retaining all the items.

This divergence in item selection can be attributed to the different theoretical assumptions underlying each applied model. CTT and IRT selected models are unidimensional, whereas the KST selected model is not. It is worth noting that CTT and IRT differ profoundly in how they formalize the notion of “dimension” and how they link items to this dimension. In CTT, the factor loadings just express the strength of the relationship between each item and the latent factor. In addition, IRT provides information on the location of items on the latent trait with respect to respondents’ abilities and, in the 3-PL case, also on the probability of guessing the correct response by chance. To obtain such fine-grained information, IRT models need a set of stronger assumptions than the CTT models. This may explain why the unifactorial solution obtained within the CTT allows for retaining almost all original items, while that obtained with the 3-PL discards more than half of them.

KST adopts a multidimensional perspective formalized through the so-called knowledge structures. This perspective allows it to retain items that cannot be located along a unidimensional continuum but remain informative for characterizing individual performance. The knowledge structure is the distinctive feature of KST that does not exist in CTT and IRT. The powerful characteristic of knowledge structures is that they provide a very precise hypothesis about the structure of relationships among items or between items and underlying skills, which is then subjected to empirical validation. In the other two approaches, the specific structure of the relationships among items or between items and underlying skills can only be derived a posteriori, after data analysis. In other words, the specific form of the construct under measurement is theory-driven in KST, whereas it is mostly data-driven in the other two approaches. This has to be seen as an advantage of KST with respect to the other two approaches (see, e.g., Fried, 2020), especially with respect to predictive power. Indeed, KST can be viewed as a complementary validation approach, as it relies on an explicit theory-based process in which items are constructed, and their connections are identified in advance, guided by cognitive theories of knowledge organization and learning progression.

Given the unique and detailed information provided by KST at the end of an assessment, and given the novelty of the theory, this section concludes with an illustrative example based on the assessment outcomes of three respondents from the sample. The aim is to highlight the differences between KST and the other two approaches. The three respondents are referred to as John, Sophie, and Jane. John and Sophie have 0 schooling years and are 4.79 and 6.24 years old, respectively. Jane has 5 schooling years and is 11.31 years old. Both John and Sophie obtained a sum score of 18 out of 38 (exhibiting different response patterns), whereas Jane achieved a sum score of 30 out of 38. Based on these scores, CTT would indicate that John and Sophie exhibit the same level of fluid intelligence, and that both perform at a lower level than Jane. Since John and Sophie displayed different response patterns, the 3-PL model is able to distinguish between their levels of fluid intelligence based on the different item discriminations and pseudoguessing. Nonetheless, their performance is reduced to a single numerical value summarizing their fluid intelligence levels.

Conversely, KST provides fine-grained information. Specifically, it provides detailed feedback on three key aspects: (i) the cardinality $|\hat{K}|$ of the individual’s knowledge state and that of the competence state $|\hat{C}|$; (ii) the number of careless errors and lucky guesses; and, most importantly, (iii) which transformation rules belong to the competence state of the individual.

Tables 12 and 13 provide a comparative overview of the three respondents’ performance. The first table summarizes the quantitative indices derived from KST, whereas the second table presents the corresponding qualitative information, highlighting differences in the transformation rules mastered by each respondent. From the KST perspective, John masters ten items, committed one careless error, and made nine lucky guesses. In contrast, Sophie masters 15 items, made two careless errors and four lucky guesses. Finally, Jane masters 33 items, and made three careless errors. Based on their knowledge states, Jane made more careless errors than Sophie who, in turn, made more careless errors than John. For lucky guesses, the opposite order was observed. This nuanced information highlights how KST can offer a more comprehensive evaluation of an individual’s performance by also considering their error tendencies.

**Table 12:** KST-based comparison of the performance of John (0 years of schooling, 4.79 years old), Sophie (0 years of schooling, 6.24 years old), and Jane (5 years of schooling, 11.31 years old)

	John	Sophie	Jane
|*R*|	18	18	30
$|\hat{C}|$	5	7	11
$|\hat{K}|$	10	15	33
$P(\hat{K})$	.96	.97	.87
# $\beta _q$	1	2	3
# $\eta _q$	9	4	0

Peculiar information provided by KST on the specific cognitive tasks mastered by each individual is relevant for formulating a diagnostic report that goes beyond the mere numerical score. For the example at hand, it could go along the following lines. Jane masters all 11 rules, demonstrating a complete grasp of the entire set of cognitive tasks. These rules include complex transformations and logical operations, indicating a high level of cognitive flexibility and problem-solving ability. In contrast, John and Sophie mastered five and seven rules, respectively (Table 13). The rules mastered by John are completion, shape, O-addition, O-subtraction, and V-logic, indicating a partial understanding of visuospatial transformations and mastery of elementary directional logic rules only. Sophie, on the other hand, masters completion, orientation, filling, shape, O-addition, O-subtraction, and V-logic, reflecting a broader understanding, especially of visuospatial transformation rules, than John.

**Table 13:**
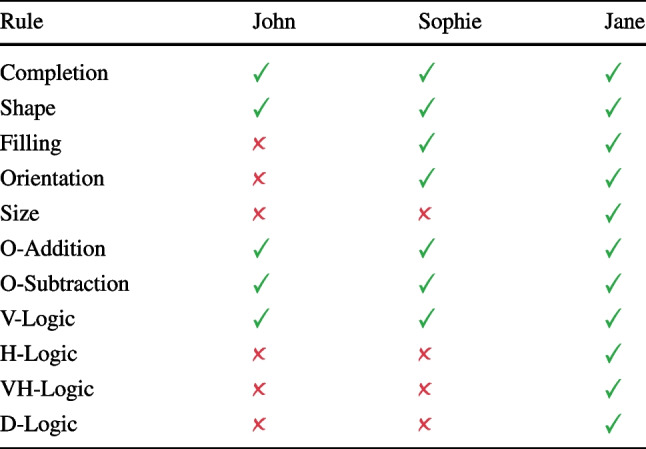
Comparison of the mastered transformation rule profiles of the three real respondents: John (0 years of schooling, 4.79 years old), Sophie (0 years of schooling, 6.24 years old), and Jane (5 years of schooling, 11.31 years old)

This example underscores a crucial aspect of KST: it accounts for the complexity and diversity of individual cognitive profiles. Even with equivalent performance on a given assessment, individuals may experience and interact with the world differently, resulting in distinct profiles of rule mastery. In this case, while both John and Sophie achieve the same observed score, their underlying cognitive profiles and areas of strength and weakness differ. By recognizing and understanding these individual differences, clinicians can tailor instruction to address specific areas of need and promote more effective training.

## Discussion

This study provides evidence for the robustness and versatility of MatriKS as a measure of fluid intelligence for children in the general population aged 4 to 11 years. The validation was conducted using a multi-method approach involving three distinct measurement frameworks: CTT, IRT, and KST. Each of the three approaches with its own assumptions and models, highlights structural properties of data that are not captured by the other two approaches. Nevertheless, the three approaches provide an acceptable modeling of the data supporting the adequate functioning of MatriKS.

Specifically, CTT and IRT evaluated MatriKS primarily through statistical criteria, focusing on item functioning, score reliability, and measurement along a latent ability continuum. In contrast, KST validation relies on a theory-based perspective, modeling the relationships among items and individuals’ response patterns with respect to an explicit cognitive representation of the underlying structure of fluid intelligence. Importantly, these approaches also differ in the type of feedback they provide: CTT and IRT models yield summative information about overall performance, whereas KST offers structured feedback on individual cognitive profiles. When considered jointly, these measurement perspectives may contribute to a more comprehensive and informative assessment by combining robust measurement properties with theory-driven insights into individual performance.

Furthermore, the obtained results – such as the consistent increase in average fluid intelligence scores among children aged 4 to 11 and the decrease in score variability across schooling years – are consistent with established literature (see, e.g., Horn, 2007; Schroeders et al., 2015). These findings provide compelling evidence supporting the external validity of the test. Moreover, the multi-method validation approach sets a benchmark for the development of psychological and neuropsychological tests. While this study establishes the validity and robustness of MatriKS, several avenues for future research and development remain to be explored.

First, future studies should explore the application of MatriKS in clinical populations. This would involve testing its reliability and validity among children with various cognitive, neurodevelopmental, or learning disabilities. Understanding how MatriKS performs in these groups could provide valuable insights into its diagnostic utility and potential modifications needed to accommodate specific clinical needs.

Second, an adaptive version of MatriKS could be developed, which adjusts the administration of subsequent questions based on the responses to the previous ones. This approach reduces time and frustration for the test-takers. Moreover, adaptivity allows clinicians to administer the test to individuals who, otherwise, could be excluded due to their clinical conditions.

Finally, exploring how MatriKS can be integrated into training programs to monitor and support children with various cognitive disabilities represents another valuable direction. The multi-method feedback provided by MatriKS, which identifies both strengths and areas for improvement, makes it a robust tool for both assessment and training design. In particular, the KST-like fine-grained feedback provided by MatriKS represents a valuable tool for supporting the clinician in developing personalized paths of interventions. The ultimate goal is to provide each child with tailored support for their cognitive development.

## Supplementary Information

Below is the link to the electronic supplementary material.

Supplementary file 1 (pdf 50 KB)

## Data Availability

The data used for the analyses are available on the Open Science Framework at: https://osf.io/7n4x2/?view_only=1c67d6d579774601aec7dddc5c5cc7c9.

## Code availability

The code used for the analyses are available on the Open Science Framework at: https://osf.io/7n4x2/?view_only=1c67d6d579774601aec7dddc5c5cc7c9.
