# Supplementary material for: Multi-method validation of the new computerized test of fluid intelligence MatriKS
Source: Behav Res Methods. 2026 Jun 8;58(7):191. doi: 10.3758/s13428-026-03049-2 (PMC13246548; doi:10.3758/s13428-026-03049-2)
Supplement: Supplementary file 1 — (pdf 50 KB) [file 13428_2026_3049_MOESM1_ESM.pdf]

Supplementary material for the paper entitled "Multi-method validation of the new computerized test of fluid intelligence MatriKS"

Table 1 shows the distribution of participants across different school levels.

Table 1: Participants' distribution by school level

**School level**	**n**	**%**
Kindergarden		
Second year	29	12
Third year	31	13
Primary School		
First year	40	17
Second year	12	5
Third year	41	17
Fourth year	59	25
Fifth year	25	11
